# Supplementary material for: The causal role of circulating inflammatory markers in osteoporosis: a bidirectional Mendelian randomized study
Source: Front Immunol. 2024 Jul 18;15:1412298. doi: 10.3389/fimmu.2024.1412298 (PMC11291241; doi:10.3389/fimmu.2024.1412298)
Supplement: Supplementary file 1 [file DataSheet_1.zip › supplementary material/CIM-OSTEOPOROSIS /GCST90274807.csv_forest.pdf]

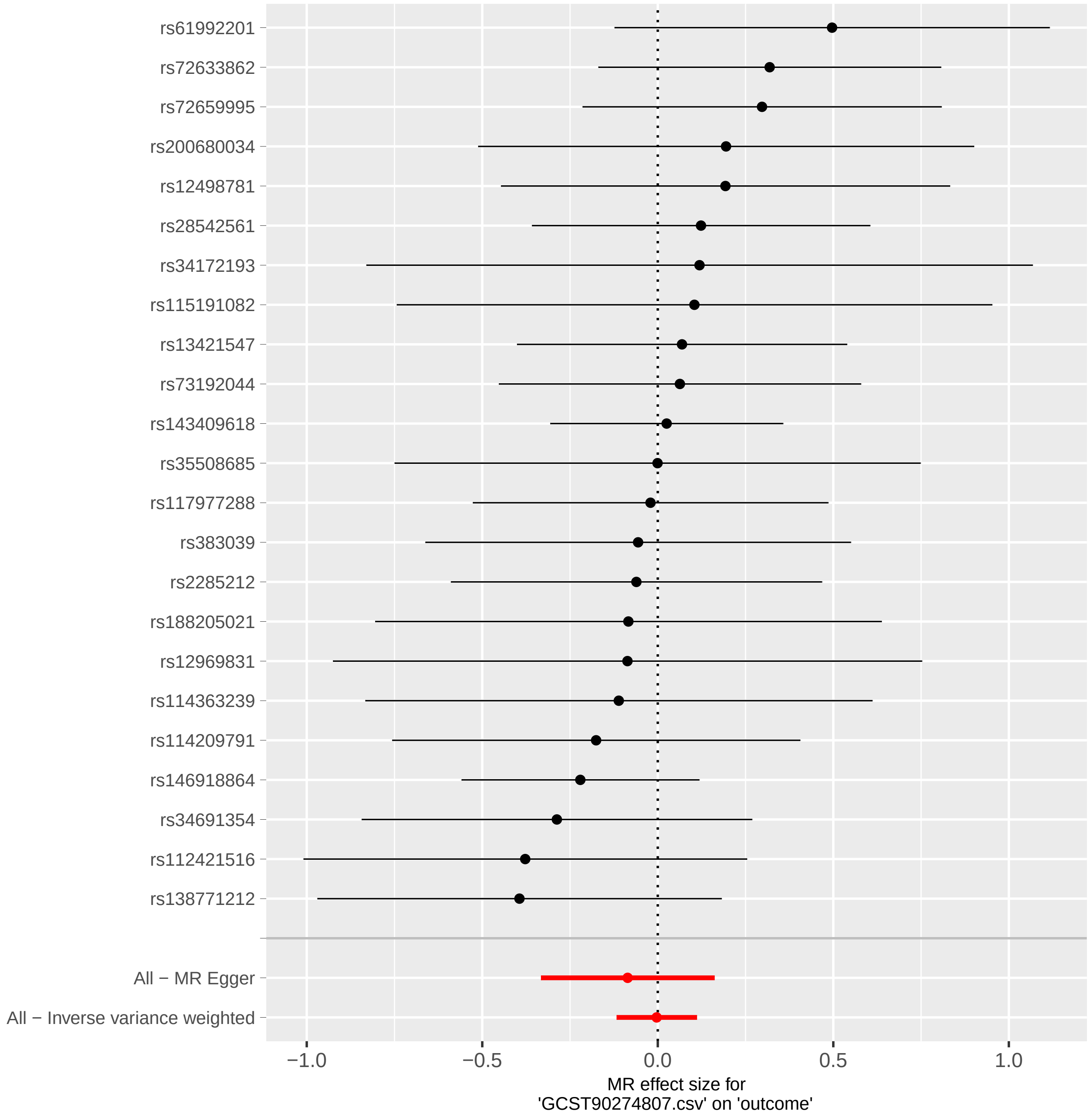
rs61992201
rs72633862
rs72659995
rs200680034
rs12498781
rs28542561
rs34172193
rs115191082
rs13421547
rs73192044
rs143409618
rs35508685
rs117977288
rs383039
rs2285212
rs188205021
rs12969831
rs114363239
rs114209791
rs146918864
rs34691354
rs112421516
rs138771212
All − MR Egger
All − Inverse variance weighted
−1.0
−0.5
0.0
0.5
1.0
MR effect size for
'GCST90274807.csv' on 'outcome'
